# A paradox in individual-based models of populations

**DOI:** 10.1093/conphys/cow023

**Published:** 2016-06-22

**Authors:** Jaap van der Meer

**Affiliations:** 1 NIOZ Royal Netherlands Institute for Sea Research, Department of Coastal Systems, and Utrecht University, P.O. Box 59, 1790 AB Den Burg Texel, The Netherlands; 2 Department of Animal Ecology, VU University, Amsterdam, The Netherlands

**Keywords:** dynamic energy budget theory, individual-based models, physiologically structured population models

## Abstract

The standard dynamic energy budget model is widely used to describe the physiology of individual animals. It assumes that assimilation rate scales with body surface area, whereas maintenance rate scales with body volume. When the model is used as the building block of a population model, only limited dynamical behaviour, the so-called juvenile-driven cycles, emerges. The reason is that in the model juveniles are competitively superior over adults, because juveniles have a higher surface area-to-volume ratio. Maintenance requirements for adults are therefore relatively large, and a reduced assimilation rate as a result of lowered food levels will easily become insufficient. Here, an alternative dynamic energy budget model is introduced that gives rise to adult-driven cycles, which may be closer to what is often observed in reality. However, this comes at the price of a rather odd description of the individual, in that maintenance scales with body area and assimilation rate with body volume, resulting in unbounded exponential body growth. I make a plea to solve the paradox and come up with reliable descriptions at both the individual and the population level.

## Abstract (precis)

Population models in ecology are nowadays often based on what we know on the physiology of individuals. I claim that a paradox exists between what we know about individuals and populations. I make a plea to get it right at both levels.

## Introduction

The scope of conservation physiology includes the development of mechanistic relationships between population declines and physiological processes (Cooke *et al.*, 2013) or, as Metcalfe *et al.* (2012) stated, ‘conservation physiology is the study of physiological responses of organisms to environmental changes and human-induced impacts, and their implications for population … dynamics’. One example concerns the impact that increased temperatures, as a result of climate change, might have on the reproductive development of Pacific salmonids during their freshwater migration and, consequently, on recruitment and stock size (Young *et al.*, 2006).

To deduce population phenomena from physiological processes at the individual level is, in fact, a general challenge in ecology, with many hidden pitfalls that are not widely recognized. I believe that a major gap still exists between what we observe at the level of the individual and what we know about population dynamics. Almost all models of individual physiology, when used as a building block in population models, will lead to so-called juvenile-driven cycles, which is a type of population dynamics that is not very often observed in nature. The basic reason is that the scaling coefficient for assimilation rate is generally assumed to be lower than the scaling rate for maintenance rate. The paradox is thus that descriptions at the individual level do not directly lead to reliable descriptions at the population level. I will illustrate my point by confronting the theoretical framework of population ecology of ontogenetic development, worked out by de Roos and Persson (2013), with that of dynamic energy budget (DEB) theory, developed by Kooijman (Kooijman, 1993, 2010).

de Roos and Persson (2013) show that the most basic information on the type of population dynamics one can expect is to be found in what they call ontogenetic asymmetry. By this, they mean that animals of different size are not equally efficient in acquiring food or in the use of assimilated energy. The ontogenetic asymmetry is best illustrated by the critical resource density R* vs. body size function, where R* is defined as the resource density at which energy intake is just enough to pay metabolic demands and at which somatic body growth equals zero. Ontogenetic symmetry is obtained only when the mass-specific rate at which new biomass is produced does not depend upon body size. When the critical resource density R* increases with body size, small individuals (e.g. juveniles) have a competitive advantage over larger individuals (e.g. adults). This occurs, for example, when assimilation rate scales with body area and maintenance rate with body volume. The opposite is true when R* decreases with body size. de Roos and Persson (2013) discuss in chapter 10 of their book a specific case where all animals have only one shared resource. Reproduction occurs as a discrete event at the start of each season. By varying one of the parameters of the function that relates intake rate to resource density, either juveniles or adults obtain competitive advantage. Juvenile advantage gives rise to a true single-cohort cycle driven by recruits, which means that each newborn cohort of recruits almost immediately depresses the resource density to such a low level that their parent cohort dies from starvation. The newborns will mature, reproduce and in turn be wiped out by their progeny. Adult advantage yields an entirely different type of cycle. New cohorts will not always be able to survive at the ambient food level. Only when the size of the dominant adult cohort decreases as a result of background mortality or ageing, with increasing resource levels as a result, do newborns have a chance to settle and mature into adulthood.

The DEB theory, developed by Kooijman (Kooijman, 1993, 2010), is the most comprehensive theory that links the environment to the major physiological processes of individual organisms, including those processes that are directly relevant for population dynamics, such as feeding, growth, reproduction and survival. The inclusion of reserves acting as metabolic memory, the full life cycle of individuals (embryo, juvenile and adult) and the explicit use of conservation laws (energy, chemical elements and isotopes) sets the DEB theory apart from other approaches. Over the last few years, a wealth of published [see, for example van der Meer *et al.* (2014) and references therein for recent contributions] and unpublished (compiled at www.bio.vu.nl/thb/deb) parameter estimations of the DEB model were done for a rapidly growing number of species. This collection of species, which comes under the name of the add_my_pet collection, has ∼400 entries at present. Almost all larger animal phyla are represented, and all chordate classes. Within DEB theory, the individual is considered the basic unit and its metabolism forms the basis of population dynamics. Much less attention has been paid to the population dynamics of individuals that follow the standard DEB model (Kooi and van der Meer, 2010).

The first aim here is to show what type of dynamics emerges from the standard DEB model, without additional assumptions at the individual level. Second, for illustrative purposes and for contrast, a rather unusual variant of the DEB model will be introduced. This peculiar model has opposite scaling relationships to the standard DEB model. The behaviour of the two models at the individual and at the population level will be compared and discussed, with the ideas on, for example, resource density functions, as provided by de Roos and Persson (2013), in mind. It will become clear (which may not come as a surprise to many readers) that the two models have very different resource density functions and population behaviour. The standard DEB model gives rise to juvenile-driven cycles and the alternative to adult-driven ones. But they also have very different behaviour at the individual level.

The environment in which the populations occur is a so-called semi-chemostat, in which the inflowing food concentration is constant. Pulsed reproduction, with a constant period in between two reproductive events, is assumed as in Kooi and van der Meer (2010). Apart from death by starvation, additional background mortality is added. Given that the background mortality level strongly determines the type of dynamics, I perform a bifurcation analysis of this parameter.

In short, after an introduction of the individual models and the semi-chemostat model environment, some general model predictions at the population level will be shown and discussed in light of resource density functions. Finally, the physiological background of juvenile–adult competition will be discussed, and a plea for more detailed physiological studies will be made.

## Materials and methods

### Models for the individual

Below, I will give a short introduction to the standard DEB model for the individual organism and to its peculiar variant. A more extensive introduction is given in the Appendix, but I refer the reader also to Kooijman (2010) for a detailed description of underlying DEB assumptions and derivations or to van der Meer (2006), who provides a more easily accessible introduction.

The organism has three succeeding life stages, as follows: the embryo, which neither feeds nor reproduces; the juvenile, which feeds but does not reproduce; and the adult, which feeds and reproduces. The organism is described by three state variables: (i) structural body volume; (ii) reserve density, which is the amount of reserves per unit of structural body volume; and (iii) maturity, which is the cumulative energy allocated to development. Embryos and juveniles develop, i.e. they build up maturity. Transitions between embryo and juvenile and between juvenile and adult occur at fixed levels of maturity. Once the animal has become adult, it has reached its maximal maturity and starts to reproduce. In this study, I assume that the adult builds up a reproduction buffer, which is emptied at the end of each reproductive period. The sum of maturity and reproduction buffer are considered here as a single state variable.

A list of assumptions give rise to a set of coupled ordinary differential equations for the three state variables. Assumptions for the standard DEB model are, among other things, that (i) assimilation rate is proportional to the surface area of the structural body; (ii) all assimilated energy enters the reserves and is then mobilized from the reserves (the rate of changes of the reserves is thus the difference between the assimilation rate and the mobilization rate); (iii) a fixed fraction, *κ*, of the mobilization rate is spent on maintenance, which is assumed to be proportional to structural body volume, and on growth, assuming fixed costs for growth per unit volume; and (iv) the rate of change of maturity equals 1 − *κ* times the mobilization rate minus the maturity maintenance costs, which are proportional to maturity.

The standard DEB model can be entirely rewritten in a dimensionless form; that is, all state variables and time are scaled by some quantity that has the same physical dimension as the original variable. For example, structural body length (the cubic root of structural body volume) is scaled by maximal body length. Such scaling has the advantage that the equations look much simpler, and the dynamical behaviour of the system of coupled differential equations (i.e. the equations for reserve dynamics, growth and maturity/reproduction) can be more easily studied without any loss of generality. The dynamics of the scaled reserve density, *e*, in scaled time, *τ*, are given by:

(1) $$\frac{de}{dt}=\frac{f-e}{,}$$

where *f* is the so-called scaled functional response that relates the assimilation rate to the food density, and takes a value between zero (no food) and one (*ad libitum*). Note that food density is the only environmental variable. Growth is given by the differential equation for scaled length, *l*:

(2) $$\frac{dl}{dt}=\frac{1}{e+g}\frac{e-l}{3},$$

where the compound parameter *g* is called the ‘energy investment ratio’. It stands for the energetic costs of new structural volume relative to the maximal energy within the reserves that is available for growth and maintenance. The sum of maturity and reproduction buffer is, after scaling, given by:

(3) $$\frac{de_{H}+e_{R}}{dt}=(1-k)\left(\frac{e}{e+g}l^{2}(g+l)-\min(l^{3},l_{p}^{3})\right),$$

where *l*_p_ is the scaled length at puberty, i.e. at the transition from juvenile to adult.

**Figure 1:**
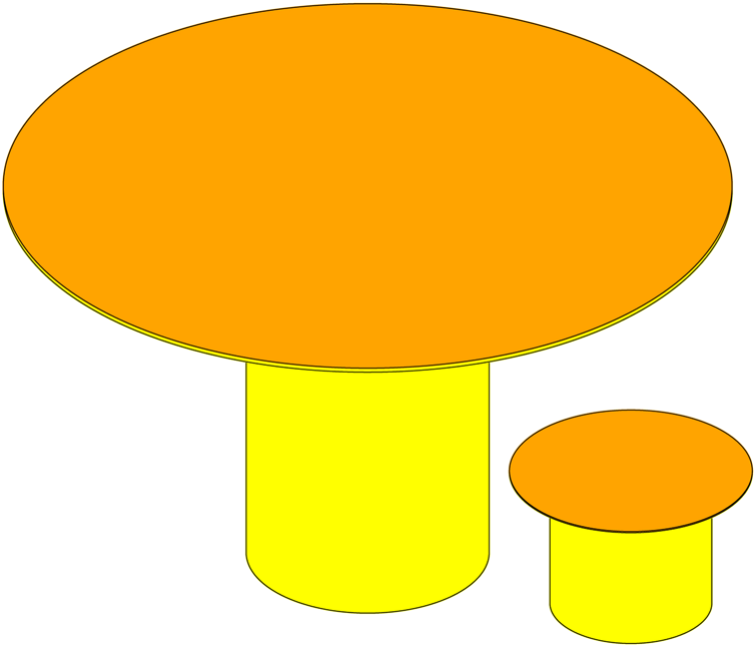
The weird animal, for which the uptake rate of food is proportional to the area of the orange body part. Maintenance rate is proportional to the area of the yellow-coloured body proper. The surface area of the orange appendix is proportional to the volume of the yellow body proper.

Now assume some peculiar animal that has a specific appendix by which it takes up resources. Uptake (and assimilation) rate is proportional to the surface area of this appendix, and this surface area is proportional to the volume of the body proper. Maintenance costs, in contrast, are proportional to the surface area of the body proper. The animal is depicted at different body sizes in Fig. 1. The dynamics of the first two dimensionless state variables, scaled reserve density *e* and scaled length *l*, are described by:

(4) $$\frac{de}{dt}=f-e,$$

and

(5) $$\frac{dl}{dt}=\frac{el-1}{3(e+g)}.$$

Note that the length at equilibrium at constant *f* (l* = 1/*f*) can no longer be interpreted as the maximal length, as for the standard DEB model. A better interpretation is the theoretical minimal length of a juvenile. If *e* = 1 at hatching, then *l* should at least be larger than 1 in order to obtain a positive length growth rate.

Finally, the dimensionless form of the differential equation for maturity plus reproduction is as follows:

(6) $$\frac{de_{H}+e_{R}}{dt}=(1-k)\frac{e}{e+g}(gl^{3}+l^{2})-\min(e_{H}+e_{R},e_{H}^{p}),$$

where $e_{H}^{p}$ is the maximal cumulative energy invested in maturity. The predicted growth curve for the animal is rather peculiar. When scaled length becomes much larger than 1, length growth rate becomes proportional to length. In other words, the body grows exponentially, until infinity or for as long food suffices, for which reason I call this animal the weird one.

### The semi-chemostat

The semi-chemostat is a well-mixed environment and has a continuous inflow and outflow of food items. The dilution rate, which is defined as the flow rate in volume per time divided by the volume of the chemostat, is constant. The organisms do not flow out, contrary to what is usually assumed in chemostats; hence, the term semi-chemostat. It is further assumed that the organisms are iteroparous and reproduce at regular intervals, all at the same time. This is sometimes called pulsed reproduction (Tang and Chen, 2002). For convenience, I call the interval between reproductive events a year. The population thus consists of clearly separated cohorts. All individuals within each cohort are exactly the same. They are born at the same time, grow and develop at the same rate, and reproduce at the same time. I assume that each animal can live for at most 3 years, after which it reproduces for the last time and dies from ageing. Other reasons for death are starvation, or to be more precise, I assume that an individual dies when *κ* times the mobilization rate drops below the required maintenance costs. Finally, I assume a constant background mortality rate, *µ*.

The system can thus be represented by a set of 13 differential equations, four equations for each of the three cohorts plus one extra equation for the food level. The four equations per cohort refer to scaled length (equation 2 or 5), scaled reserve density (equation 1 or 4), scaled maturity/reproduction (equation 3 or 6) and the number of individuals. The differential equation for the number of individuals per cohort, *i*, is as follows:

(7) $$\frac{dn_{i}}{dt}=-mn_{i}.$$

Within each reproductive season (or year), several events may happen. The individuals within a cohort may be born (the transition from embryo to juvenile), which means that feeding starts and *f* changes from 0 to some non-zero value. They may mature (the transition from juvenile to adult), which implies that a start is made with filling the reproduction buffer, or they may die from starvation. During integration, these state-dependent switches are checked.

The differential equation for scaled food density, *x*, is given for DEB animals by:

(8) $$\frac{dx}{dt}=h(x_{0}-x)-\sum_{i=1}^{3}n_{i}ql_{i}^{2}f,$$

where *x*_0_ is the scaled food density of the inflowing water, *h* the scaled dilution rate, and *q* the scaled relative ingestion rate. For the weird individuals, the same equation is used, but with $l_{i}^{3}$ replacing $l_{i}^{2}.$ Underlying scaling factors also differ.

At the end of each year, integration stops. The sum of the reproduction buffers $\left(\Sigma_{i=1}^{3}n_{i}e_{R,i}l_{i}^{3}\right)$ determines the size of the new cohort for the next integration period. See Kooi and van der Meer (2010) for mathematical details and a rigorous stability analysis. Parameter values used are given in Table 1. The choice of these values does not qualitatively affect the results.

**Table 1:** Parameter values of the semi-chemostat model for the standard dynamic energy budget individual and the weird individual

Symbol	Interpretation	Dynamic energy budget	Weird
*g*	Maintenance rate coefficient	1	1
*κ*	Fraction of mobilization rate spent on maintenance plus growth	0.8	0.8
*κ*_R_	Reproductive efficiency	0.95	0.95
$e_{H}^{b}$	Scaled maturity at birth	–	0.25
$e_{H}^{p}$	Scaled maturity at puberty	–	6
*l*_b_	Scaled length at birth	0.16	–
*l*_p_	Scaled length at puberty	0.6	–
*l*_0_	Initial scaled length	0.001	0.001
*e*_0_	Initial scaled reserve density	$(1+g+3/4l_{b})l_{b}^{3}/l_{0}^{3}$	$5.75/l_{0}^{3}$
$e_{H}^{0}$	Initial scaled maturity	$(1-k)gl_{0}^{3}$	0
*x*_0_	Scaled food input	5	500
*q*	Scaled maximal feeding rate	0.01	10^−8^
*h*	Scaled dilution rate	0.1	0.1
*τ*_R_	Length of the reproductive period in scaled time	10	40
*µ*	Background mortality	0.01–0.25	0.01–0.35

## Results

**Figure 2:**
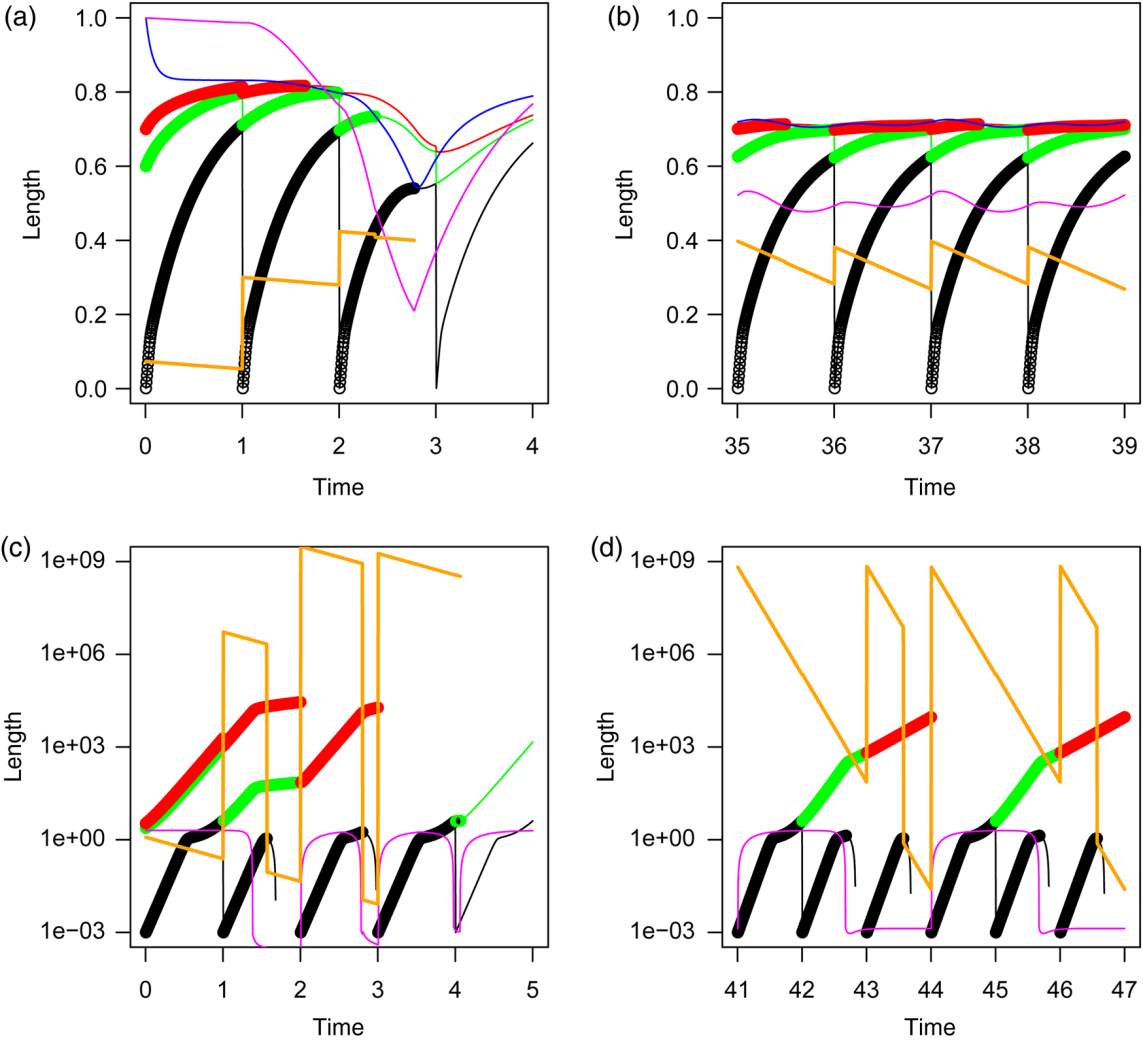
Length (black dots for the first year, green for the second year and red for the third year of life), reserve density (continuous blue line) and food levels (continuous magenta line) and population size (thick continuous orange line, no axis) vs. time in years. The thin continuous black, green and red lines show length if the animals would not have died (but would neither have affected food levels any more). Upper left *µ* = 0.03 (**a**), upper right *µ* = 0.17 (**b**), both for the standard DEB model. The two lower panels (**c** and **d**) refer to the weird animal. Left for *µ* = 0.04 (c) and right for *µ* = 0.2 (d).

**Figure 3:**
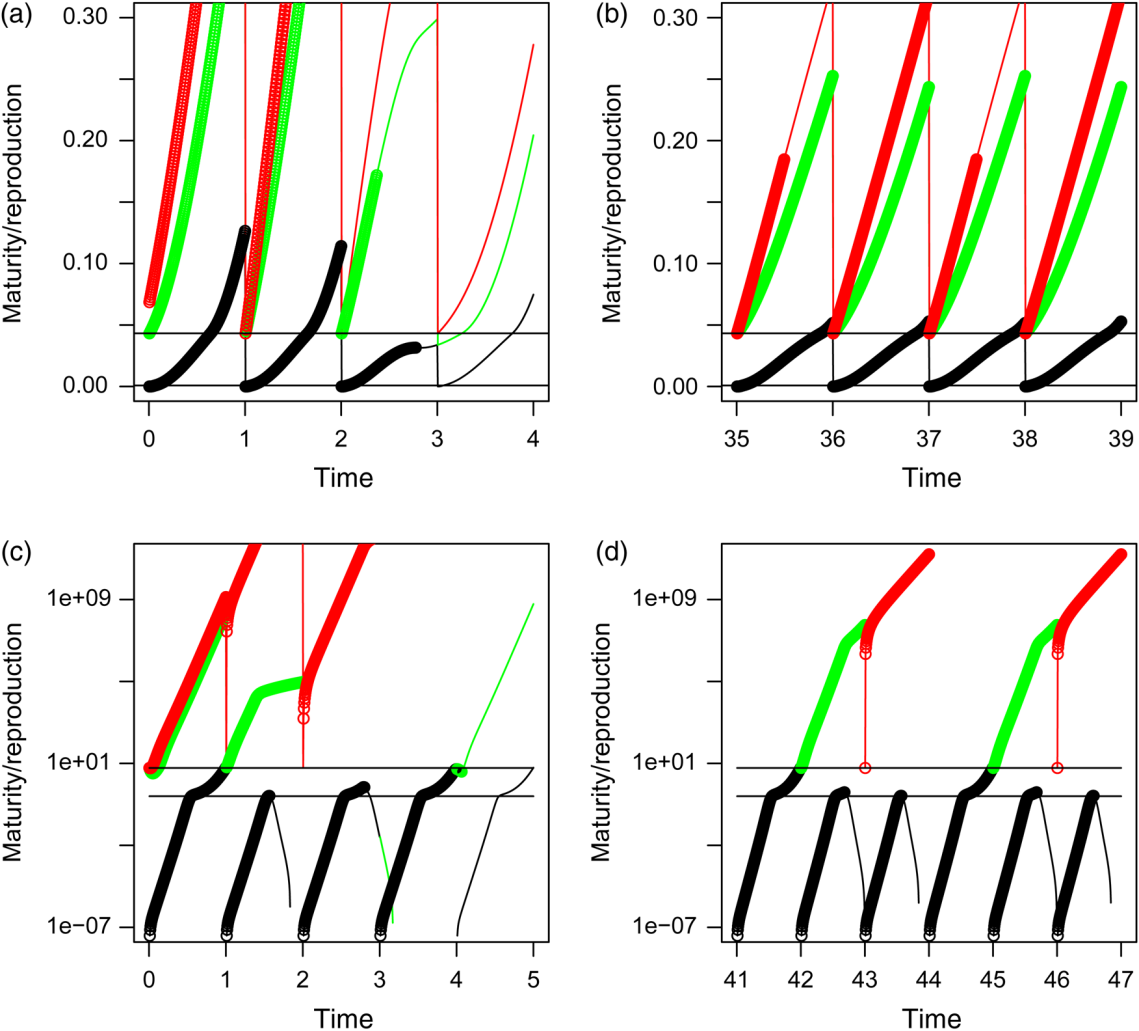
Maturity plus reproduction buffer (black dots for the first year, green for the second year and red for the third year of life) vs. time in years. The thin continuous black, green and red lines show maturity/reproduction if the animals would not have died (but would neither have affected food levels any more). The two lower panels (**c** and **d**) refer to the weird animal. Upper left *µ* = 0.03 (**a**), upper right *µ* = 0.17 (**b**), both for the standard DEB model. The two lower panels (**c** and **d**): left for *µ* = 0.04 (c) and right for *µ* = 0.2 (d). Horizontal black lines indicate the threshold maturity at birth and at puberty. Note that at the end of a year, the reproduction buffer, if present, is emptied to zero. Maturity plus reproduction buffer then equals the threshold maturity at puberty.

The semi-chemostat model population of DEB individuals started with a low number of individuals, distributed over three cohorts. The first cohort consisted of newly laid eggs with initial state *e*_0_, *l*_0_ and $e_{H}^{0},$ as given in Table 1, and the other two cohorts are adults with, for both cohorts, scaled reserve density *e* equal to 1, scaled length *l* equal to 0.6 for the first and 0.8 for the second adult cohort, and scaled maturity *e*_H_ = (1−*κ*)*gl*^3^ equal to 0.04 and 0.10, respectively. At a low background mortality, *µ*, the population increases quickly at each reproductive event, up to the situation when the youngest cohort, containing the smallest individuals, is so large that it depletes the food resource down to a level where the largest individuals can no longer pay their maintenance and die. Figure 2a shows a situation where this event happened halfway through the second year. When the continuous blue line, indicating the reserve density, *e*, of the largest animals, drops below the line through the red dots, which shows the scaled length, *l*, of the same animals, the animals die. In other words, when *e* < *l* the animals can no longer pay their maintenance and die. Shortly after the beginning of the third year, the oldest cohort dies, quickly followed by the death of the second cohort and even before the end of the same year by the death of the cohort of newborns itself. Food level gets so low that growth and maturation (Fig. 3a) are severely retarded. The last cohort dies even before it matures, and the population goes extinct.

**Figure 4:**
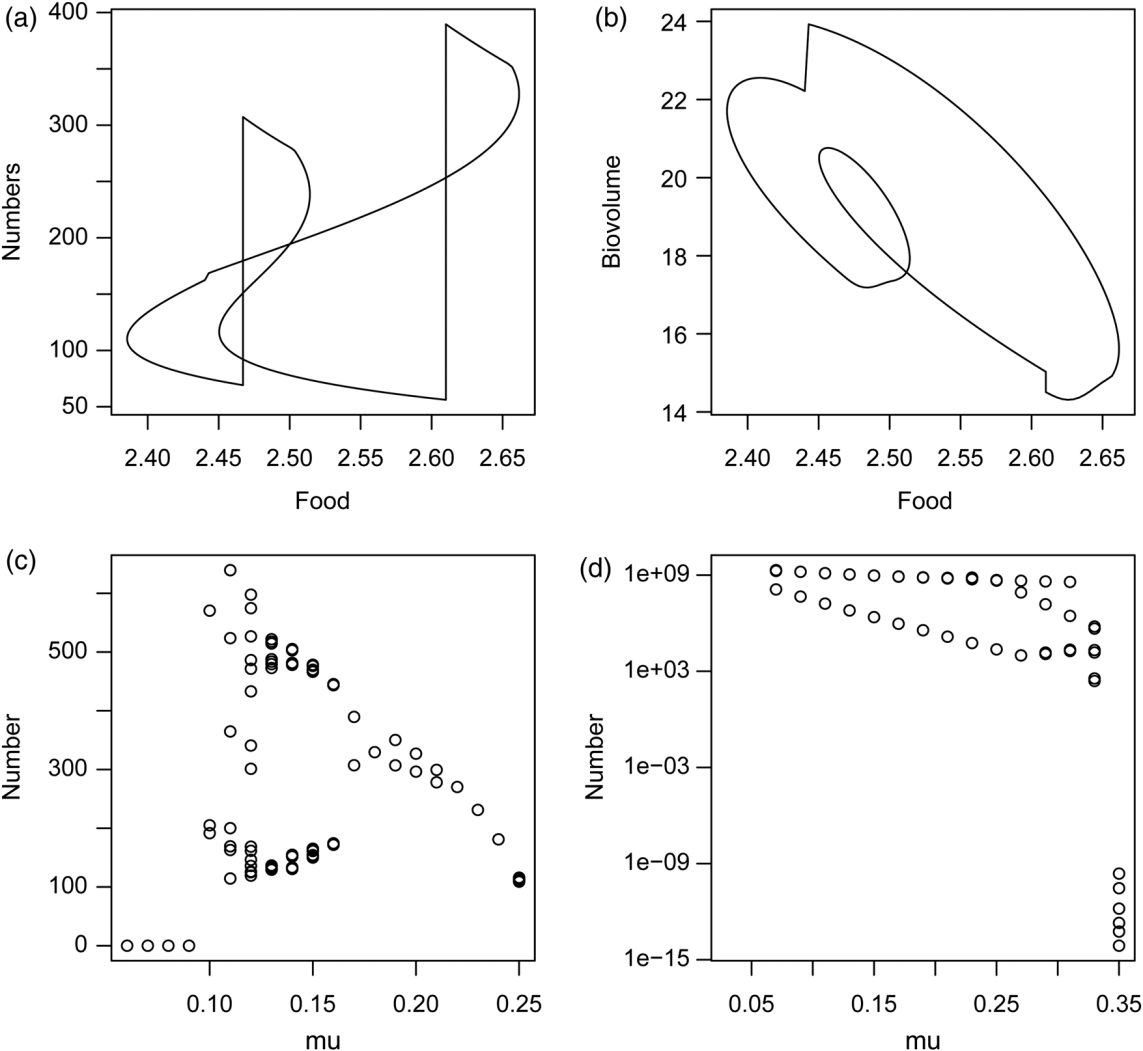
Food vs. number of animals (**a**) and food vs. total biovolume of all animals (**b**), for the standard DEB model with *µ* = 0.17. (**c** and **d**) Total number of animals at the start of the season vs. background mortality *µ* for the standard DEB model (c) and the weird animal (d).

Stable periodic solutions occur only with larger background mortalities, as visualized by the bifurcation diagram of Fig. 4c. A stable period-*n* cycle (or period-*n* attractor) means a solution that repeats itself every *n*th year. With increasing background mortality, *µ*, attractors with longer periods appear. For example, at *µ* = 0.10, a period-3 attractor, and at *µ* = 0.12 even a period-16 attractor is observed. When mortality is higher, period-2 and period-1 attractors occur, and above *µ* = 0.25 a stable population is no longer possible. Figure 4a and b summarizes the dynamics in terms of a food–numbers and a food–biovolume phase plane diagram for *µ* = 0.17, which provides a period-2 attractor. At each reproductive period, one observes a sudden increase in numbers, initially followed by a steady increase in food level (recall that embryos do not feed) and a steady decrease in numbers. This gives rise to a more or less linear part in the trajectory through the phase plane. Shortly after hatching, when the newborns begin to feed, food level starts to drop quickly. The number of animals drops gradually (recall that background mortality is constant), but with additional discrete steps when an entire cohort dies from starvation. When enough animals have gone, food level starts to increase again until shortly after the second reproductive event. The two periods yield a similar pattern. The biovolume–food plot shows a related but different pattern. The older cohorts play a more prominent role here. For example, at the two types of reproductive events, biovolume hardly changes or even goes down as a result of the death of the oldest cohort. Biovolume may also increase when numbers decrease. Figures 2b and 3b show more details of the two-period cycle for *µ* = 0.17.

For the weird animal, the situation is completely different. At low background mortality (Figs 2c and 3c), the population goes extinct too, as in the DEB model, but for a different reason. When population size increases, which already occurs in the second year after the start of the simulation, new cohorts do not get a chance to survive after birth. Food levels are too low for them to pay their relatively high maintenance costs. Only when the oldest cohort dies from ageing do these youngsters get an opportunity. Yet, when their number is too high, food levels might drop quickly below the required level for maintenance for the newly hatched generation. The newborns die before they are able to reproduce. Population size should thus not become too high, and this occurs only at higher background mortality (Figs 2d and 3d). Then the newborn juveniles survive, and for most values of *µ* a period-2 cycle is observed. (Fig. 4d).

## Discussion

For the standard DEB model, the relationship between critical resource density expressed in terms of the scaled functional response, and scaled body length, immediately follows from equation 16: f* = *l* (assuming that the scaled reserve density, *e*, is in equilibrium with the scaled functional response, *f*, as predicted by equation 14). In the standard DEB model, *f* does not depend on the structural body volume, and follows the same strictly increasing function of resource density, whatever the size of the animal. Hence, the bigger you are, the higher the critical f* and the higher the critical resource density. The DEB juveniles are thus competitively superior over DEB adults. Basically, this observation is simply a consequence of assimilation rate being a function of surface area and maintenance rate being a function of volume; or, stated otherwise, of the scaling coefficient for assimilation rate being smaller than the scaling rate for maintenance rate. It thus holds also for many other model approaches, such as for the growth model of the metabolic theory of ecology, the so-called ontogenetic growth model (Hou *et al.*, 2008). It thus does not hold for the weird animal, for which the assimilation scaling coefficient is higher than the maintenance scaling coefficient. Indeed, equation 23 says that the critical resource density is a decreasing function of scaled body length, f* = 1/*l*.

de Roos and Persson (2013) already predict that the type of dynamics one can expect follows from the critical resource density function. Thus, it should not come as a surprise that the dynamics that I observed for the DEB animal and for the weird animal resemble the dynamics they describe in their chapter 10. But let us compare the two studies in some more detail. In the standard DEB model and in the model of my weird animal, food availability could be summarized by the scaled functional response, *f*, because *f* is independent of body size. The reason is that the searching rate (also called the attack rate) and the maximal ingestion rate (also considered as the handling rate or the inverse of the handling time) do have the same scaling coefficient with body size in these models. The model of chapter 10 of de Roos and Persson (2013) is more complicated. The maximal ingestion rate (or inverse handling time) in their model basically scales with body mass with an empirically based coefficient equal to 0.8. The maintenance rate scales with a coefficient equal to 0.75, which is lower. Their model individual thus very much resembles my weird animal. That is, in *ad libitum* food conditions (when attack rate does not play a role), the model predicts unbounded exponential length growth towards infinity. Yet, in the model of de Roos and Persson (2013), the attack rate, which becomes important at low food levels, has a scaling coefficient that is not necessarily the same as that of the maximal ingestion rate. de Roos and Persson (2013) use three different values for the scaling coefficient, 0.6, 0.8 and 1.04. In fact, the function they use for relating attack rate to body size is slightly more complicated than a simple allometric relationship and levels off at larger body size, but that is not really important for the present argument. What is important is that when the scaling coefficient for the attack rate is 0.6 and hence is much smaller than the scaling coefficient for the maintenance rate (which was 0.75), the juveniles are competitively superior. The critical resource density increases with body size, with juvenile-driven cycles as a result. The opposite is true when the attack rate scaling coefficient is much higher, i.e. 1.04, than the maintenance rate coefficient. Increasing body size then yields a lower critical resource density, and adults dominate.

By varying the scaling coefficient of the attack rate, de Roos and Persson (2013) are able to generate a range of dynamical behaviour, which cannot be predicted by the DEB model. Only by introducing a weird animal have I been able to create adult competitive superiority resulting in adult-driven cycles. Martin *et al.* (2013) used a parametrized DEB model of an individual water flea, *Daphnia magna*, as the building block of an individual-based population model. They compared the predicted population dynamics with data from a semi-batch culture experiment. The experiments started with low number of neonates and/or adults and lasted for ∼40 days (Preuss *et al.*, 2009). The experimental population size increased quickly after an initial lag phase and declined again to low numbers in the second half of each experiment. The model predicted the observations rather well during the growth phase, but not during the decline phase. The authors had to induce extra starvation of the smaller individuals to obtain a better fit with observational data at the low food levels during the decline phase. In a similar study, McCauley *et al.* (2008) also had to impose increasing starvation tolerance with size. Such adult competitive superiority is sometimes even taken for granted in ecological textbooks. For example, Paul Colinvaux, author of an ecological textbook and essays on ecology (Colinvaux, 1979), writes ‘when times are very hard through too much crowding … it is eggs, embryos and young that are starved … It is … the unfinished animal that succumbs’. Another example is the huge work of fishery biologists. For the post-recruitment phase, they generally assume a constant growth equation (usually the Bertalanffy) and a constant size-dependent reproduction, implicitly assuming constant food availability. At the same time, they assume a decelerating stock-recruitment relationship, implying that density dependence occurs only in the pre-recruitment phase. It is the pre-recruit that succumbs.

Here we have the paradox. The DEB model predicts juvenile advantage, and only the weird animal model predicts adult advantage. Students of the energetics of individual organisms have never come up with a description of the energetics that looks like that for the weird animal or for the de Roos–Persson animal. The scaling coefficient for assimilation rate is always smaller than for maintenance rate. Length growth curves always show a decelerating growth rate when the animal gets larger. This implies that adult competitive advantage should not occur. As we have seen, students of population dynamics might observe the opposite. But what then is the mechanistic, or physiological and/or behavioural if you like, explanation of adult dominance? In a theoretical study, Le Bourlot *et al.* (2014) showed that size-related interference competition in favour of the larger animals can induce a type of adult-driven cycles in a population that otherwise experienced juvenile-driven cycles. But can interference competition, or cannibalism as a more extreme form of interference, be a general explanation for adult dominance? Another explanation might be related to food quality. Perhaps juveniles require higher-quality food than adults, making a model with only one food type mostly irrelevant. Or is DEB theory missing something else at the individual level?

I would like to make a plea for detailed physiological studies on adult–juvenile differences in survival during harsh food conditions, in order to see to what extent exploitative competition for a single food resource could result in adults being competitively superior over juveniles. A better understanding of adult–juvenile competition is urgently needed for individual-based population modelling, where both the individual and the population level are adequately described. A challenge for conservation physiologists!

## Appendix

Further details and derivations are given for the following: (i) the standard DEB model for the individual organism; (ii) the weird animal; and (iii) the chemostat model.

### The standard dynamic energy budget model

The standard DEB model has three state variables: structural body volume, *V*; reserve density, [*E*] = *E*/*V*, which is the amount of reserves per unit of structural body volume; and maturity, *E*_H_, plus reproduction buffer, *E*_R_, which is the cumulative energy allocated to development and reproduction. Thus, embryos and juveniles develop (build up maturity), and adults reproduce (build up a reproduction buffer). The sum of the latter two paramters, *E*_H_ plus *E*_R_, is considered as a single state variable (Table 2). The most important environmental variable is the food density, *X* (Table 2).

**Table 2:** State variables of the standard dynamic energy budget model and environmental variables

Symbol	Dimension	Interpretation
*V*	*L*^3^	Structural body volume
[*E*]	*eL*^−3^	Reserve density
*E*_H_ + *E*_R_	*e*	Maturity plus reproduction buffer
*T*	*T*	Temperature
*X*	#*l*^−3^	Food density in the environment

Abbreviations: *L* stands for the dimension length of the structural body, *e* for energy, # for mass measured in terms of moles of Carbon, and *l* for the dimension length of the environment.

A set of assumptions, e.g. that assimilation rate is proportional to the surface area of the structural body, ${\dot{p}}_{A}=\{{\dot{p}}_{\mathrm{AM}}\}fV^{2/3},$ and others referring to specific types of homeostasis, give rise to the ordinary differential equation for reserve density:

(9) $$\frac{d[E]}{dt}=V^{-1/3}(\{{\dot{p}}_{Am}\}f-\dot{v}[E]).$$

The area-specific assimilation rate, $\{{\dot{p}}_{\mathrm{AM}}\},$ and the energy conductance, $\dot{v},$ are so-called primary parameters of the standard DEB model. They are explained, together with quite a few others, such as $\left\{{\dot{F}}_{m}\right\}$ and *κ*_X_, in Table 3; *f* is the so-called scaled functional response that relates the assimilation rate to the food density, and is given by:

$$f=\frac{X}{\{{\dot{p}}_{Am}\}/(\{{\dot{F}}_{m}\}m_{X}k_{X})+X},$$

and *µ*_X_ is the chemical potential of the food.

**Table 3:** Primary parameters of the standard dynamic energy budget model

Symbol	Dimension	Interpretation	Process
$\left\{{\dot{p}}_{\mathrm{AM}}\right\}$	*eL*^−2^*t*^−1^	Surface-area-specific maximal assimilation rate	Assimilation
$\left\{{\dot{F}}_{m}\right\}$	*l*^3^*L*^−2^*t*^−1^	Surface-area-specific searching rate	Feeding
*κ*_X_	–	Digestion efficiency	Digestion
$\dot{v}$	*Lt*^−1^	Energy conductance	Mobilization
*κ*	–	Fraction of mobilization rate spent on maintenance plus growth	Allocation
$\left[{\dot{p}}_{M}\right]$	*eL*^−3^*t*^−1^	Volume-specific maintenance rate	Turnover/activity
$\left\{{\dot{p}}_{T}\right\}$	*eL*^−2^*t*^−1^	Surface-area-specific maintenance rate	Heating/osmosis
[*E*_G_]	*eL*^−3^	Volume-specific costs of growth	Growth
${\dot{k}}_{J}$	–	Specific maturity maintenance	Regulation/defence
*κ*_R_	–	Reproductive efficiency	Egg formation
$E_{H}^{b}$	*e*	Maturity at birth	Life history
$E_{H}^{p}$	*e*	Maturity at puberty	Life history

See the legend of Table 2 for an explanation of the abbreviations.

The growth equation, assuming that there are no heating costs involved, is given by:

(10) $$\frac{dV}{dt}=\frac{k\dot{v}[E]V^{2/3}-[{\dot{p}}_{M}]V}{k[E]+[E_{G}]}.$$

It follows from:

(11) $$\frac{dE}{dt}=\frac{d[E]V}{dt}={\dot{p}}_{A}-{\dot{p}}_{C},$$

which tells us that the rate of change of the reserves is the difference between the assimilation rate, ${\dot{p}}_{A},$ and the mobilization rate, ${\dot{p}}_{C}.$ A fraction, *κ*, of the mobilization rate is spent on maintenance, which is assumed to be proportional to structural body volume, and on growth, assuming fixed costs for growth per unit volume. Using this so-called *κ*-rule and the product rule for differentiation, equation 11 can be rewritten as follows:

$$V\frac{d[E]}{dt}+[E]\frac{dV}{dt}=\{{\dot{p}}_{\mathrm{Am}}\}fV^{2/3}-\frac{1}{k}[{\dot{p}}_{M}]V-\frac{1}{k}[E_{G}]\frac{dV}{dt}.$$

Combination with equation 9 and some re-arrangement yields the growth equation, equation 10.

The rate of change of maturity equals 1 − *κ* times the mobilization rate minus the maturity maintenance costs, which are proportional to maturity. Hence:

(12) $$\frac{dE_{H}}{dt}=(1-k){\dot{p}}_{C}-{\dot{k}}_{J}E_{H},$$

for $E_{H}\;<\;E_{H}^{p}$; otherwise, that is when the animals have become mature and $E_{H}=E_{H}^{p},$ maturity does not change any more and d*E*_H_/d*t* = 0.

In this study, I assume that once the animal has become mature, or in other words once it has turned into an adult, it starts to build up a reproduction buffer. The rate of change of the reproduction buffer is given by:

(13) $$\frac{dE_{R}}{dt}=(1-k){\dot{p}}_{C}-{\dot{k}}_{J}E_{H}^{p},$$

for $E_{H}=E_{H}^{p}.$ I further assume that at the end of each reproductive period, when *t* = *t*_R_, the buffer is emptied and *E*_R_ is set back to 0. The reproduction, i.e. the number of progeny after each reproductive period, equals:

$$R=k_{R}\frac{E_{R}(t_{R})}{E_{0}},$$

where *κ*_R_ is the reproductive efficiency and *E*_0_ the initial energy content of an egg. It can be shown that the mobilization rate, ${\dot{p}}_{C},$ equals:

$${\dot{p}}_{C}=\frac{\left[E\right]}{k[E]+[E_{G}]}(\dot{v}[E_{G}]V^{2/3}+[{\dot{p}}_{M}]V).$$

The system of differential equations for reserve density (equation 9), structural volume (equation 10) and maturity/reproduction (equations 12 and 13) describe the standard DEB model.

The standard DEB model can be entirely rewritten in a dimensionless form. In order to arrive at a dimensionless model, one has to rescale all dimensions, i.e. energy, length (or volume) and time. The choice of scaling coefficients is rather arbitrary, as we will see. Yet, for energy an obvious choice is the maximal amount of energy in reserve, *E*_m_, and for volume it is the maximal volume of the structural body, $V_{m}=(k\{{\dot{p}}_{\mathrm{AM}}\}/[{\dot{p}}_{M}]{)}^{3}.$ The maximal amount of energy in reserve equals the product of the maximal reserve density and the maximal volume, *E*_m_ = [*E*_m_]*V*_m_, where $[E_{m}]=\{{\dot{p}}_{\mathrm{Am}}\}/\dot{v}$ as follows from equation 9 with *f* = 1. These choices ensure that the new dimensionless state variables, which are scaled reserve density, *e* ≡ [*E*]/[*E*_m_], and scaled length, *l* ≡ (*V*/*V*_m_)^1/3^, are easy to interpret and to remember. The same holds for scaled maturity, *e*_H_ ≡ *E*_H_/*E*_m_. The choice of a scaling coefficient for time is less obvious, but it helps to look first at power, which is given as energy per time. One option is to scale power by the maximal assimilation rate, which equals ${\dot{p}}_{\mathrm{Am}}=\{{\dot{p}}_{\mathrm{Am}}\}V_{m}^{2/3}.$ This choice implies that time is scaled to *κ* times the ratio of the maximal reserve density and the volume-specific maintenance rate, resulting in $\tau \equiv t[{\dot{p}}_{M}]/(k[E_{m}])$.

Not only the state variables, but also all parameters (Table 4) and model equations can now be rescaled. Equation 9, which describes the dynamics of the reserve density, turns into:

(14) $$\frac{de}{dt}=\frac{d[E]}{dt}\frac{1}{\left[E_{m}\right]}\frac{k[E_{m}]}{\left[{\dot{p}}_{M}\right]}=\frac{f-e}{l}.$$

**Table 4:** Rescaling the primary parameters of the standard dynamic energy budget model written in an energy–length framework into a dimensionless framework

Energy-length	Dimension	Dimensionless
$\left\{{\dot{p}}_{\mathrm{AM}}\right\}$	*eL*^−2^*t*^−1^	1
$\dot{v}$	*Lt*^−1^	1
$\left[{\dot{p}}_{M}\right]$	*eL*^−3^*t*^−1^	*κ*
$\left\{{\dot{p}}_{T}\right\}$	*eL*^−2^*t*^−1^	*κl*_T_
[*E*_G_]	*eL*^−3^	*κg*
$E_{H}^{b}$	*e*	$e_{H}^{b}$
$E_{H}^{p}$	*e*	$e_{H}^{p}$

Energy is scaled to the maximal energy in reserves [*E*_m_]*V*_m_, volume to the maximal volume *V*_m_, and power to the maximal assimilation rate $\{{\dot{p}}_{\mathrm{Am}}\}V_{m}^{2/3}.$ Hence, time is scaled to one over the product of the energy investment ratio and the maintenance rate coefficient $(g{\dot{k}}_{M}{)}^{-1}$.

Likewise, the growth equation, equation 10, becomes:

(15) $$\frac{dl^{3}}{dt}=\frac{dV}{dt}\frac{1}{V_{m}}\frac{k[E_{m}]}{\left[{\dot{p}}_{M}\right]}=\frac{1}{e+g}l^{2}(e-l),$$

which is equivalent $\left(dl^{3}/dt=(dl^{3}/dl)\;(dl/dt)=3l^{2}dl/dt\right)$ to:

(16) $$\frac{dl}{dt}=\frac{1}{e+g}\frac{e-l}{3},$$

where the compound parameter *g* is given by the ratio [*E*_G_]/(*κ*[*E*_m_]). This is one of the most important compound parameters in DEB theory and is called the ‘energy investment ratio’. It stands for the energetic costs of new structural volume, [*E*_G_], relative to the maximal available energy for growth and maintenance, *κ*[*E*_m_]. Equations 12 and 13 are combined and rewritten as follows:

(17) $$\begin{array}{c} \frac{de_{H}\;+\;e_{R}}{dt}\;=\;\frac{dE_{H}\;+\;E_{R}}{dt}\frac{1}{[E_{m}]V_{m}}\frac{k[E_{m}]}{\left[{\dot{p}}_{M}\right]}\\ \;\;\;\;\;\;\;\;\;\;\;=\;(1\;-\;k)l^{2}\frac{e}{e+g}(g\;+\;l)\;-\;\frac{{\dot{k}}_{J}}{{\dot{k}}_{M}g}\min(e_{H}\;+\;e_{R},e_{H}^{p}), \end{array}$$

where the compound parameter ${\dot{k}}_{M},$ called the ‘maintenance rate coefficient’ is given by the ratio $[{\dot{p}}_{M}]/[E_{G}].$ It stands for the maintenance costs of structure relative to the investment. When ${\dot{k}}_{J}={\dot{k}}_{M},$ which means that the relative maintenance costs of maturity equal those of the somatic body, it can be shown that

$$E_{H}=\frac{1-k}{k}[E_{G}]V$$

or, in dimensionless form, *e*_H_ = (1 − *κ*)*gl*^3^. The main result of setting ${\dot{k}}_{J}={\dot{k}}_{M}$ is thus that maturity occurs at a fixed length, that is $e_{H}^{p}=(1-k)gl_{p}^{3}.$ The consequence is that equation 17 simplifies to:

(18) $$\frac{de_{H}+e_{R}}{dt}=(1-k)\left(\frac{e}{e+g}l^{2}(g+l)-\min(l^{3},l_{p}^{3})\right).$$

The present analysis uses this simplification.

### The weird animal

Now assume some weird animal that has a specific appendix by which it takes up resources. Uptake rate is proportional to the surface area of this appendix, and this surface area is proportional to the volume of the body proper. Maintenance costs, in contrast, are proportional to the surface area of the body proper. These costs can therefore be considered as heating costs, and the volume-specific maintenance costs are negligible. Reserve density dynamics for this animal can be described by:

(19) $$\frac{d[E]}{dt}=[{\dot{p}}_{\mathrm{Am}}]f-{\dot{k}}_{E}[E],$$

where the scaled functional response *f* is now given by:

$$f=\frac{X}{[{\dot{p}}_{\mathrm{Am}}]/([{\dot{F}}_{m}]m_{X}k_{X})+X}.$$

The dimensionless form, using the scaling relationships *e* = [*E*]/[*E*_m_] and $t=t{\dot{k}}_{E}$ and the relationship $[{\dot{p}}_{\mathrm{Am}}]={\dot{k}}_{E}[E_{m}],$ looks like this:

(20) $$\frac{de}{dt}=\frac{d[E]}{dt}\frac{1}{[E_{m}]{\dot{k}}_{E}}=f-e.$$

The growth equation follows, as before, from:

$$\frac{dE}{dt}=\frac{d[E]V}{dt}=V\frac{d[E]}{dt}+[E]\frac{dV}{dt}={\dot{p}}_{A}-{\dot{p}}_{C},$$

which here takes the form

$$\begin{array}{l} V([{\dot{p}}_{\mathrm{Am}}]f-{\dot{k}}_{E}[E])+[E]\frac{dV}{dt}=[{\dot{p}}_{\mathrm{Am}}]fV-\frac{1}{k}\{{\dot{p}}_{T}\}V^{2/3}\\ \;\;\;\;\;\;\;\;\;\;\;\;\;\;\;\;\;\;\;\;\;\;-\frac{1}{k}[E_{G}]\frac{dV}{dt}, \end{array}$$

which results in:

(21) $$\frac{dV}{dt}=\frac{k{\dot{k}}_{E}[E]V-\{{\dot{p}}_{T}\}V^{2/3}}{k[E]+[E_{G}]}.$$

This growth equation can also be written in a dimensionless form, using the earlier mentioned scaling and *l* = *L*/*L*_m_, as:

(22) $$\frac{dl^{3}}{dt}=\frac{dV}{dt}\frac{L_{m}^{3}}{{\dot{k}}_{E}}=\frac{el-1}{e+g}l^{2},$$

where *L*_m_ is, as for the standard DEB model, defined as the length for which the growth rate of a well-fed animal is zero. The dimensionless length growth equation is now:

(23) $$\frac{dl}{dt}=\frac{el-1}{3(e+g)}.$$

Note that

$$L_{m}=V_{m}^{1/3}=\frac{\left\{{\dot{p}}_{T}\right\}}{k{\dot{k}}_{E}[E_{m}]},$$

can no longer be interpreted as the maximal length. A better interpretation is the theoretical minimal length of a juvenile. If *e* = 1 at hatching, then *l* should at least be larger than 1 in order to obtain a positive length growth rate. The differential equation for maturity plus reproduction is as in the standard DEB model:

(24) $$\frac{dE_{H}+E_{R}}{dt}=(1-k){\dot{p}}_{C}-{\dot{k}}_{J}\min(E_{H}+E_{R},E_{H}^{p}),$$

but the mobilization rate is now given by:

$${\dot{p}}_{C}=\frac{\left[E\right]}{k[E]+[E_{G}]}({\dot{k}}_{E}[E_{G}]V+\{{\dot{p}}_{T}\}V^{2/3}).$$

In dimensionless form it looks like this:

$$\frac{{\dot{p}}_{C}}{[{\dot{p}}_{\mathrm{Am}}]V_{m}}=\frac{e}{e+g}(gl^{3}+l^{2}),$$

and the dimensionless form of the differential equation for maturity plus reproduction becomes:

(25) $$\frac{de_{H}+e_{R}}{dt}=(1-k)\frac{e}{e+g}(gl^{3}+l^{2})-\frac{{\dot{k}}_{J}}{{\dot{k}}_{E}}\min(e_{H}+e_{R},e_{H}^{p}).$$

The assumption that ${\dot{k}}_{J}={\dot{k}}_{E}$ is made for the rest of the paper (Table 5).

**Table 5:** Extra parameters of the model for the weird individual

Symbol	Dimension	Interpretation	Process
$\left[{\dot{p}}_{\mathrm{AM}}\right]$	*eL*^−3^*t*^−1^	Volume-specific maximal assimilation rate	Assimilation
$\left[{\dot{F}}_{m}\right]$	*l*^3^*L*^−3^*t*^−1^	Volume-specific searching rate	Feeding
${\dot{k}}_{E}$	*t*^−1^	Specific energy conductance	Mobilization

The predicted growth curve for the weird animal is rather peculiar. When scaled length becomes much larger than 1, length growth rate becomes proportional to length; in other words, the body grows exponentially, until infinity or for as long food suffices, for which reason I called this animal the weird one.

### The semi-chemostat

The differential equation for food density, *X*, expressed as the number of food items per volume, is given by:

(26) $$\frac{dX}{dt}=\dot{h}(X_{0}-X)-\frac{1}{A}\sum_{i=1}^{3}n_{i}{\dot{J}}_{X,i},$$

where *X*_0_ is the food density of the inflowing water, *A* the volume of the chemostat, and ${\dot{J}}_{X,i}$ the feeding rate of an individual of cohort *i*. The feeding rate of a DEB individual with volume *V* is given by:

$${\dot{J}}_{X}=\{{\dot{J}}_{\mathrm{Xm}}\}V^{2/3}f,$$

where $\left\{{\dot{J}}_{\mathrm{Xm}}\right\}$ is the maximal surface-area-specific feeding rate. When multiplied by the assimilation efficiency, *κ*_X_, and the chemical potential of the food, *µ*_X_, it equals the maximal surface-area-specific assimilation rate $\{{\dot{p}}_{\mathrm{AM}}\},$ i.e. $\{{\dot{p}}_{\mathrm{Am}}\}=k_{X}m_{X}\{{\dot{J}}_{\mathrm{Xm}}\}.$ The scaled functional response is *f* = *X*/(*X*_K_ + *X*), where the half-saturation coefficient, *X*_K_, equals $\{{\dot{J}}_{\mathrm{Xm}}\}/\{{\dot{F}}_{m}\}.$ A weird individual feeds at a rate ${\dot{J}}_{X}=[{\dot{J}}_{\mathrm{Xm}}]Vf,$ and the half-saturation coefficient, *X*_K_, equals $\left[{\dot{J}}_{\mathrm{Xm}}]/[{\dot{F}}_{m}\right]$.

A dimensionless DEB version looks, after scaling the food density with the half-saturation coefficient (*x* = *X*/*X*_K_) and scaling time as before,

$$\frac{dx}{dt}=\frac{dX}{dt}\frac{\left\{{\dot{J}}_{\mathrm{Xm}}\right\}}{\left\{{\dot{F}}_{m}\right\}}\frac{k[E_{m}]}{\left[{\dot{p}}_{M}\right]},$$

like this:

(27) $$\frac{dx}{dt}=h(x_{0}-x)-\sum_{i=1}^{3}n_{i}ql_{i}^{2}f,$$

with scaled dilution rate $h=\dot{h}k[E_{m}]/[{\dot{p}}_{M}],$ scaled relative ingestion rate $q=\{{\dot{F}}_{m}\}V_{m}/(A\dot{v})$ and scaled functional response *f* = *x*/(1 + *x*). For the weird individuals it is:

(28) $$\frac{dx}{dt}=h(x_{0}-x)-\sum_{i=1}^{3}n_{i}ql_{i}^{3}f,$$

with $h=\dot{h}/[{\dot{k}}_{E}]$ and $q=[{\dot{F}}_{m}]V_{m}/(A{\dot{k}}_{E})$.
